# Differentiating malignant from benign salivary gland lesions: a multiparametric non-contrast MR imaging approach

**DOI:** 10.1038/s41598-021-82455-2

**Published:** 2021-02-02

**Authors:** Koji Takumi, Hiroaki Nagano, Hidehiko Kikuno, Yuichi Kumagae, Yoshihiko Fukukura, Takashi Yoshiura

**Affiliations:** Department of Radiology, Kagoshima University Graduate School of Medical and Dental Sciences, 8-35-1 Sakuragaoka, Kagoshima, 890-8544 Japan

**Keywords:** Head and neck cancer, Cancer imaging

## Abstract

The purpose of this study is to determine whether multiparametric non-contrast MR imaging including diffusion-weighted imaging (DWI), arterial spin labeling (ASL), and amide proton transfer (APT) weighted imaging can help differentiate malignant from benign salivary gland lesions. The study population consisted of 42 patients, with 31 benign and 11 malignant salivary gland lesions. All patients were evaluated using DWI, three-dimensional pseudo-continuous ASL, and APT-weighted imaging on 3 T MR imaging before treatment. Apparent diffusion coefficient (ADC), tumor blood flow (TBF), and APT-related signal intensity (APTSI) values within the lesion were compared between the malignant and benign lesions by Mann–Whitney *U* test. For each parameter, optimal cutoff values were chosen using a threshold criterion that maximized the Youden index for predicting malignant lesions. The performance of ADC, TBF, APTSI, individually and combined, was evaluated in terms of diagnostic ability for malignant lesions. Diagnostic performance was compared by McNemar test. APTSI was significantly higher in malignant lesions (2.18 ± 0.89%) than in benign lesions (1.57 ± 1.09%, p = 0.047). There was no significant difference in ADC or TBF between benign and malignant lesions (p = 0.155 and 0.498, respectively). The accuracy of ADC, TBF, and APTSI for diagnosing malignant lesions was 47.6%, 50.0%, and 66.7%, respectively; whereas the accuracy of the three parameters combined was 85.7%, which was significantly higher than that of each parameter alone (p = 0.001, 0.001, and 0.008, respectively). Therefore, the combination of ADC, TBF, and APTSI can help differentiate malignant from benign salivary gland lesions.

## Introduction

It is important to preoperatively differentiate between benign and malignant salivary gland lesions because this information influences the surgical planning. Total parotidectomy with radiotherapy is the method of choice in malignant salivary tumors, following which facial nerve may be lost. On the other hand, for benign tumors, superficial parotidectomy with only a part of facial nerve removal is selected. In addition, Warthin tumors can be handled with enucleation or conservative management because of their low potential for malignancy compared to pleomorphic adenomas and malignant tumors. Salivary gland lesions have several histological subtypes^1^, which makes it difficult to diagnose them with radiological imaging. Dynamic contrast-enhanced MR imaging has been reported to help diagnose salivary gland lesions^2^. However, previous studies have shown substantial overlaps in dynamic contrast enhancement patterns between benign and malignant salivary gland lesions^2,3^. In addition, contrast-enhanced MR imaging requires the injection of contrast material, and thus it cannot be performed in patients with renal dysfunction and allergic history. Furthermore, it leads to both prolonged scan times and increased costs.

Some non-contrast MR imaging techniques have been reported to provide useful information to diagnose salivary gland lesions^4–9^. Yabuuchi et al. reported that the apparent diffusion coefficient (ADC) of pleomorphic adenomas on diffusion-weighted image (DWI) was significantly higher than that of malignant lesions^4^. However, ADC alone cannot allow differentiation of malignant from benign lesions because of overlap in ADC between Warthin tumors and malignant lesions^4,5^. Arterial spin labeling (ASL) is a noninvasive perfusion MR imaging technique that uses the intrinsic spin state of arterial blood water as a tracer for quantitatively measuring tumor blood flow (TBF), which has been available for head and neck region^10,11^. Recently, TBF has been reported to be useful for diagnosing parotid gland lesions, especially differentiation of Warthin tumors from other parotid gland lesions^12–14^. However, one previous study^14^ reported that no significant difference in TBF was found between pleomorphic adenomas and malignant lesions. Amide proton transfer (APT)-weighted imaging is a new MR imaging technique that provides molecular information^15–18^. Recent studies have shown the usefulness of APT-related signal intensity (APTSI) in differentiating malignant from benign head and neck lesions with malignant lesions tending to have higher APTSI^19–22^, while considerable overlap in APTSI was seen between benign and malignant lesions.

We hypothesized that the combination of these three non-contrast MR imaging techniques may improve the diagnostic performance in differentiating salivary gland lesions. Therefore, the purpose of this study was to determine whether the combination of ADC, TBF and APTSI can help increase accuracy in differentiating malignant from benign salivary gland lesions.

## Results

**Figure 1:**
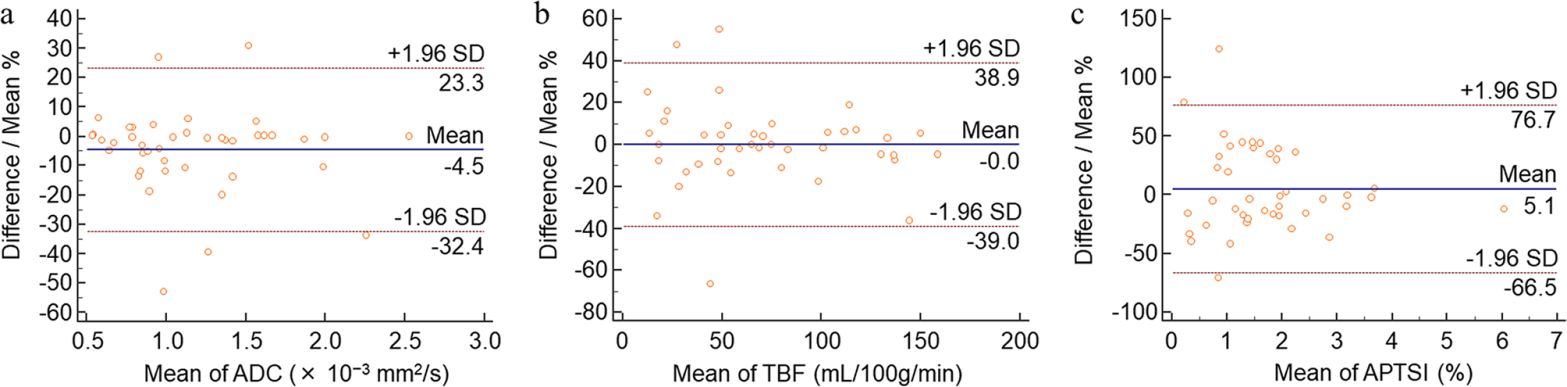
Bland–Altman plots of apparent diffusion coefficient (ADC) (**a**), tumor blood flow (TBF) (**b**), and amide proton transfer related signal intensity (APTSI) (**c**) assessing the interobserver agreement.

Interobserver agreements were excellent for all quantitative measurements (Table 1). Bland–Altman analyses showed relatively small bias and 95% limits of agreement for each quantitative parameter (Table 1, Fig. 1).

**Table 1:** Interobserver agreement.

Quantitative parameters	ICC	Bland–Altman analysis (%)		
		Bias	95% limits of agreement	
			Lower	Upper
ADC (× 10^−3^ mm^2^/s)	0.96 (0.93, 0.98)	− 4.5	− 32.4	23.3
TBF (mL/100 g/min)	0.97 (0.96, 0.99)	0	− 39.0	38.9
APTSI (%)	0.96 (0.92, 0.98)	5.1	− 66.5	76.7

Numbers in parentheses are 95% confidence intervals.

ADC, apparent diffusion coefficient; TBF, tumor blood flow; APTSI, amide proton transfer related signal intensity.

**Figure 2:**
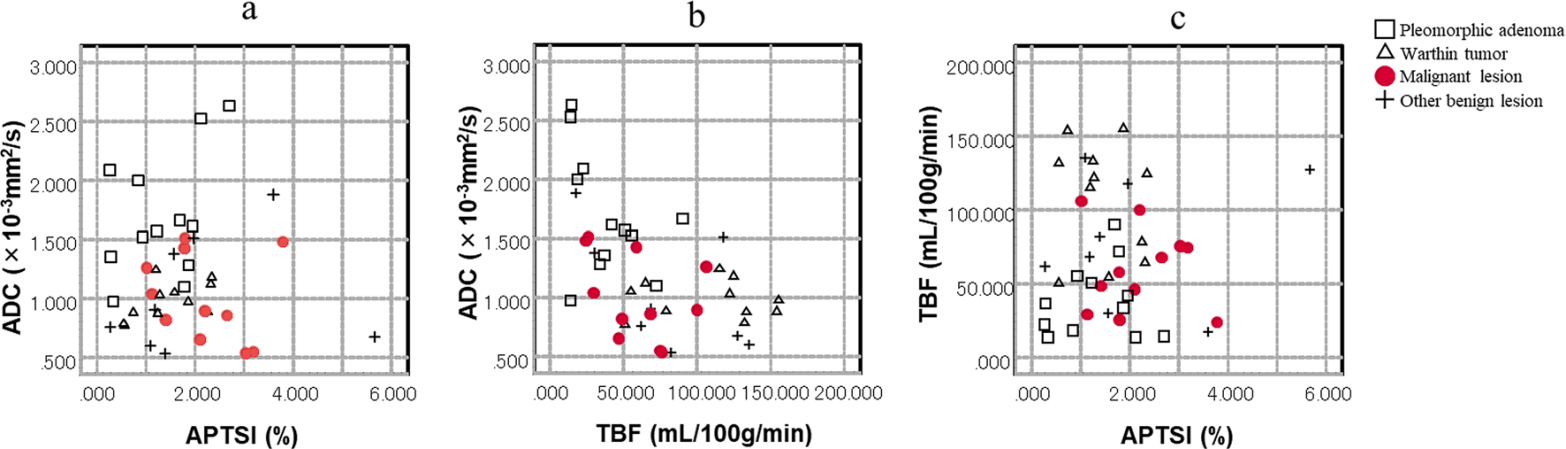
Scatterplot of ADC versus APTSI (**a**), ADC versus TBF (**b**), and TBF versus APTSI (**c**) of salivary gland lesions. Values of pleomorphic adenoma tend to cluster in the area of high ADC and low TBF and APTSI. Values of Warthin tumors tend to cluster in the area of low ADC and APTSI and high TBF. Values of malignant lesions tend to cluster in area of low ADC and TBF and high APTSI.

APTSI in malignant lesions (2.18 ± 0.89%) was significantly higher than that in benign lesions (1.57 ± 1.09%, p = 0.047) (Table 2). There were no significant differences in ADC (1.00 ± 0.37 vs. 1.27 ± 0.53 × 10^−3^ mm^2^/s, p = 0.155) or TBF (59.79 ± 28.13 vs. 73.86 ± 45.79 mL/100 g/min, p = 0.498) between malignant and benign lesions (Table 2). For subtypes of benign lesions, pleomorphic adenoma had significant high ADC and low APTSI compared to malignant lesions (p = 0.001 and 0.037, respectively). TBF of Warthin tumor was significantly higher than that of malignant lesion (p = 0.005) (Table 2). The APTSI, ADC, and TBF values of each case are plotted in Fig. 2.

**Table 2:** Comparison of MR parameters between benign and malignant salivary gland lesions.

Parameters	Malignant (n = 11)	All benign (n = 31)	p^a^	PA (n = 12)	p^a^	WT (n = 11)	p^a^
ADC (× 10^−3^ mm^2^/s)	1.00 ± 0.37	1.27 ± 0.53	0.155	1.70 ± 0.52	0.001	0.98 ± 0.16	1.000
TBF (mL/100 g/min)	59.79 ± 28.13	73.86 ± 45.79	0.498	38.70 ± 25.68	0.059	107.70 ± 38.58	0.005
APTSI (%)	2.18 ± 0.89	1.57 ± 1.09	0.047	1.33 ± 0.81	0.037	1.44 ± 0.68	0.101

ADC, apparent diffusion coefficient; TBF, tumor blood flow; APTSI, amide proton transfer related signal intensity; PA, pleomorphic adenoma; WT, Warthin tumor.

^a^Comparisons with malignant lesions were assessed using the Mann–Whitney *U* test.

**Figure 3:**
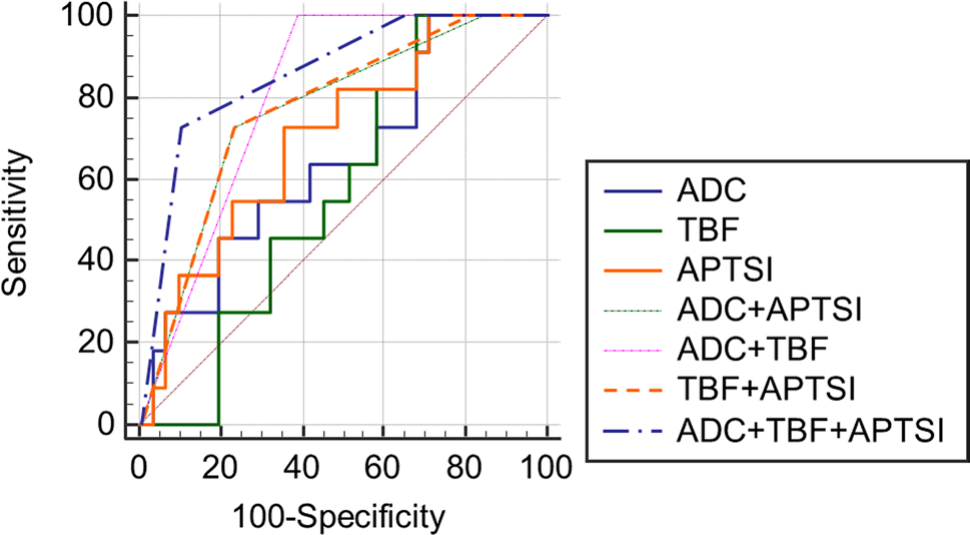
Receiver operating characteristics (ROC) curves for diagnosis of malignant salivary gland tumors. The area under the ROC curve of the combination of the three parameters (ADC, TBF, and APTSI) was significantly greater than that of each parameter alone (p = 0.044, 0.006, 0.010, respectively).

For differentiating malignant from benign lesions, the area under the ROC curve (AUC) of the combination of the three parameters (ADC, TBF, and APTSI) (0.864; 95% confidence interval, 0.722 to 0.950) was significantly greater than that of ADC alone (0.648; 95% confidence interval, 0.486 to 0.789) (p = 0.044), TBF alone (0.572; 95% confidence interval, 0.410 to 0.723) (p = 0.006), and APTSI alone (0.704; 95% confidence interval, 0.543 to 0.834) (p = 0.010) (Fig. 3). The AUC of the combination of ADC and TBF (0.806; 95% confidence interval, 0.655 to 0.912) and the combination of TBF and APTSI (0.777; 95% confidence interval, 0.622 to 0.891) were significantly greater than that of TBF alone (p = 0.013, 0.047, respectively).

The accuracy for diagnosing malignant lesion of ADC (cutoff value, ≤ 1.516 × 10^−3^ mm^2^/s), TBF (≤ 105.96 mL/100 g/min) and APTSI (> 1.779%) were 47.6%, 50.0%, and 66.7%, respectively. The combination of the two parameters (ADC with TBF, ADC with APTSI, and TBF with APTSI) had significantly higher accuracies (71.4%, 76.2%, and 76.2%, respectively) than ADC alone (p = 0.002, 0.008, and 0.029, respectively) and TBF alone (p = 0.004, 0.043, and 0.013, respectively), while their combinations had no significant differences in accuracy compared to APTSI alone (p = 0.824, 0.125, and 0.125, respectively). The accuracy of the combination of the three parameters was 85.7% which was significantly higher than that for each parameter alone (p = 0.001, 0.001, and 0.008, respectively) (Table 3).

**Table 3:** Performance of MR parameters for diagnosing malignant salivary gland lesions.

Parameters	Threshold criterion	Sensitivity (%)	Specificity (%)	PPV (%)	NPV (%)	Accuracy (%)
**Initial study (n = 42)**						
ADC (× 10^−3^ mm^2^/s)	≤ 1.516	100.0	29.0	33.3	100.0	47.6
TBF (mL/100 g/min)	≤ 105.96	100.0	32.2	34.4	100.0	50.0
APTSI (%)	> 1.779	72.7	64.5	42.1	87.0	66.7
ADC + TBF		100.0	61.2	47.8	100.0	71.4^a^
ADC + APTSI		72.7	77.4	53.3	88.9	76.2^a^
TBF + APTSI		72.7	77.4	53.3	88.9	76.2^a^
ADC + TBF + APTSI		72.7	90.3	72.7	90.3	85.7^b^
**Validation study (n = 10)**						
ADC (× 10^−3^ mm^2^/s)	≤ 1.516	100.0	29.0	37.5	100.0	50.0
TBF (mL/100 g/min)	≤ 105.96	100.0	42.9	42.9	100.0	60.0
APTSI (%)	> 1.779	66.7	57.1	40.0	80.0	60.0
ADC + TBF		100.0	71.4	60.0	100.0	80.0
ADC + APTSI		66.7	71.4	50.0	83.3	70.0
TBF + APTSI		66.7	71.4	50.0	83.3	70.0
ADC + TBF + APTSI		66.7	85.7	66.7	85.7	80.0

ADC, apparent diffusion coefficient; TBF, tumor blood flow; APTSI, amide proton transfer related signal intensity; PPV, positive predictive value; NPV, negative predictive value.

^a^Significant difference in comparison with ADC alone or TBF alone (p < 0.05).

^b^Significant difference in comparison with each parameter (ADC or TBF or APTSI) alone (p < 0.05).

Table 3 shows the results of the validation study involving 10 salivary gland lesions. When we applied the diagnostic algorithm combining the three parameters, the sensitivity, specificity, positive predictive value, negative predictive value, and accuracy were 66.7%, 85.7%, 66.7%, 85.7%, and 80.0%, respectively (Table 3). The accuracy of the combination of the three parameters improved the diagnostic values compared to each parameter alone although p value did not reach the significance level (p > 0.05, respectively).

**Figure 4:**
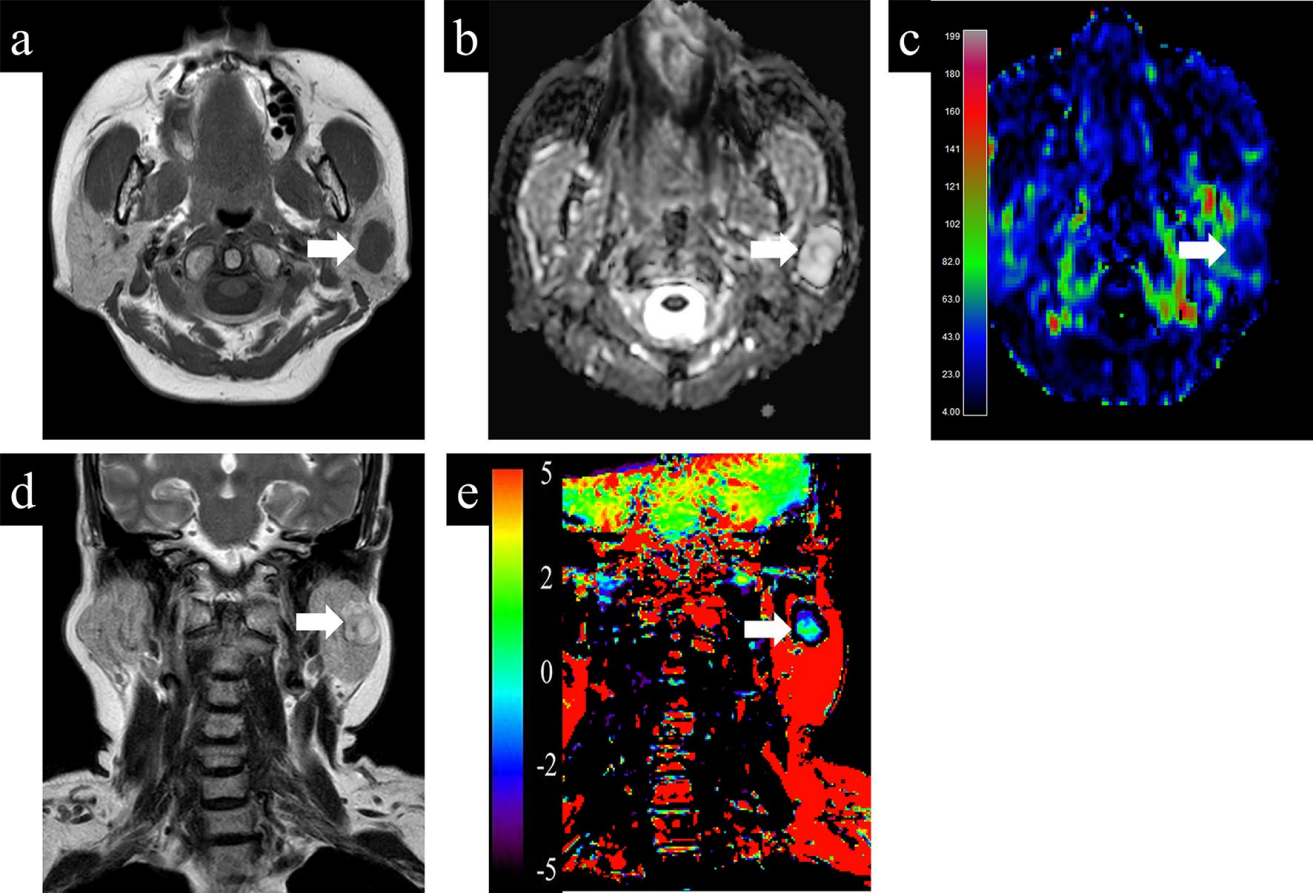
A 68-year-old female with pleomorphic adenoma. (**a**) T1-weighted image shows a homogeneous low intensity lesion in the left parotid gland. Apparent diffusion coefficient (ADC) (**b**) and tumor blood flow (TBF) (**c**) maps show the lesion with the mean ADC value of 1.88 × 10^−3^ mm^2^/s and the mean TBF value of 22.55 mL/100 g/min, respectively. On T2-weighted coronal image (**d**), the lesion shows higher signal intensity than the adjacent parotid gland parenchyma. Amide proton transfer (APT) (**e**) map shows the lesion with the mean APT-related signal intensity of 0.26%. An arrow in each image indicates the lesion.

**Figure 5:**
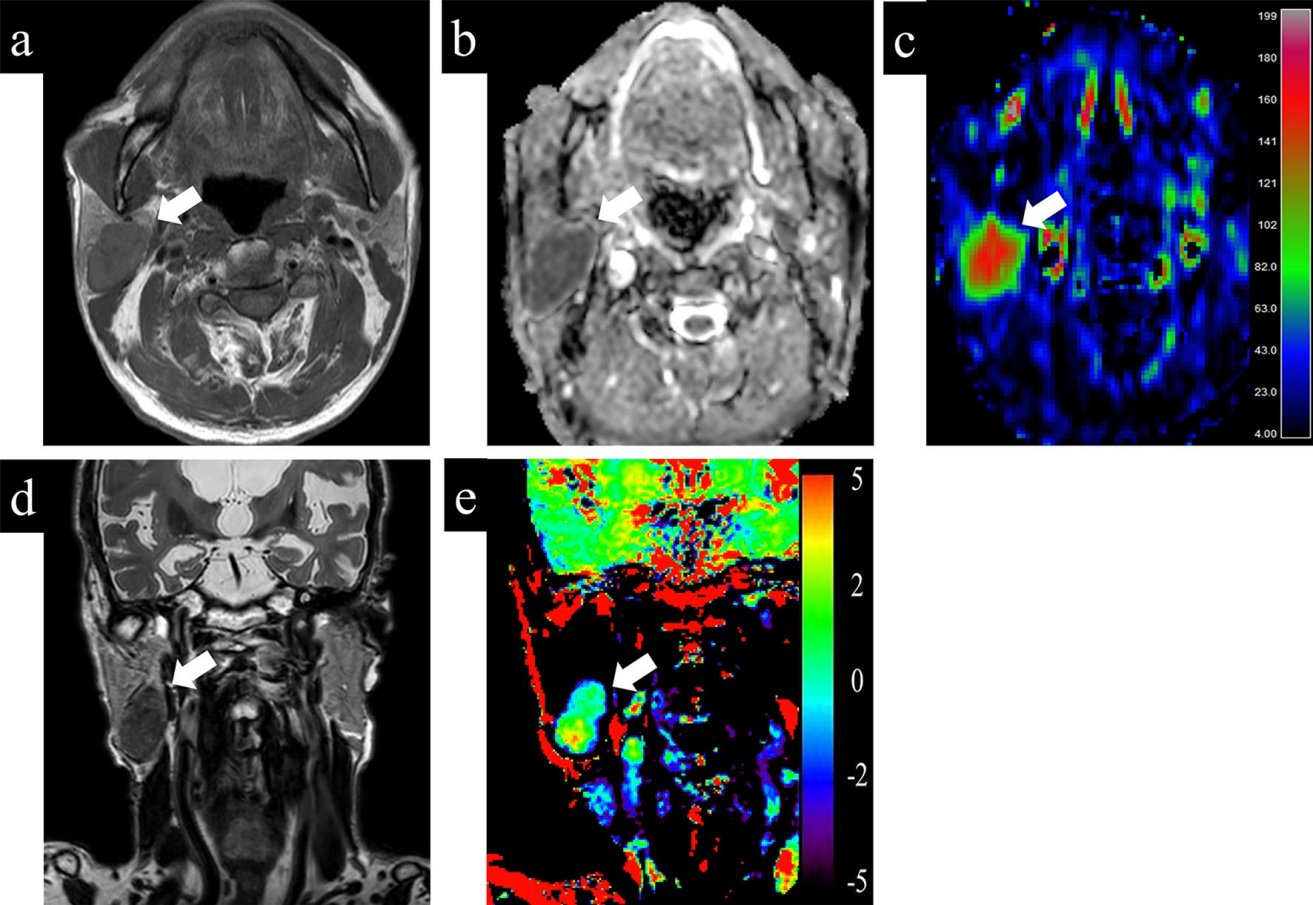
A 79-year-old male with Warthin tumor. (**a**) T1-weighted image shows a homogeneous low intensity lesion in the right parotid gland. Apparent diffusion coefficient (ADC) (**b**) and tumor blood flow (TBF) (**c**) maps show the lesion with the mean ADC value of 0.85 × 10^−3^ mm^2^/s and the mean TBF value of 133.06 mL/100 g/min, respectively. On T2-weighted coronal image (**d**), the lesion shows lower signal intensity than the adjacent parotid gland parenchyma. Amide proton transfer (APT) (**e**) map shows the lesion with the mean APT-related signal intensity of 1.24%. An arrow in each image indicates the lesion.

**Figure 6:**
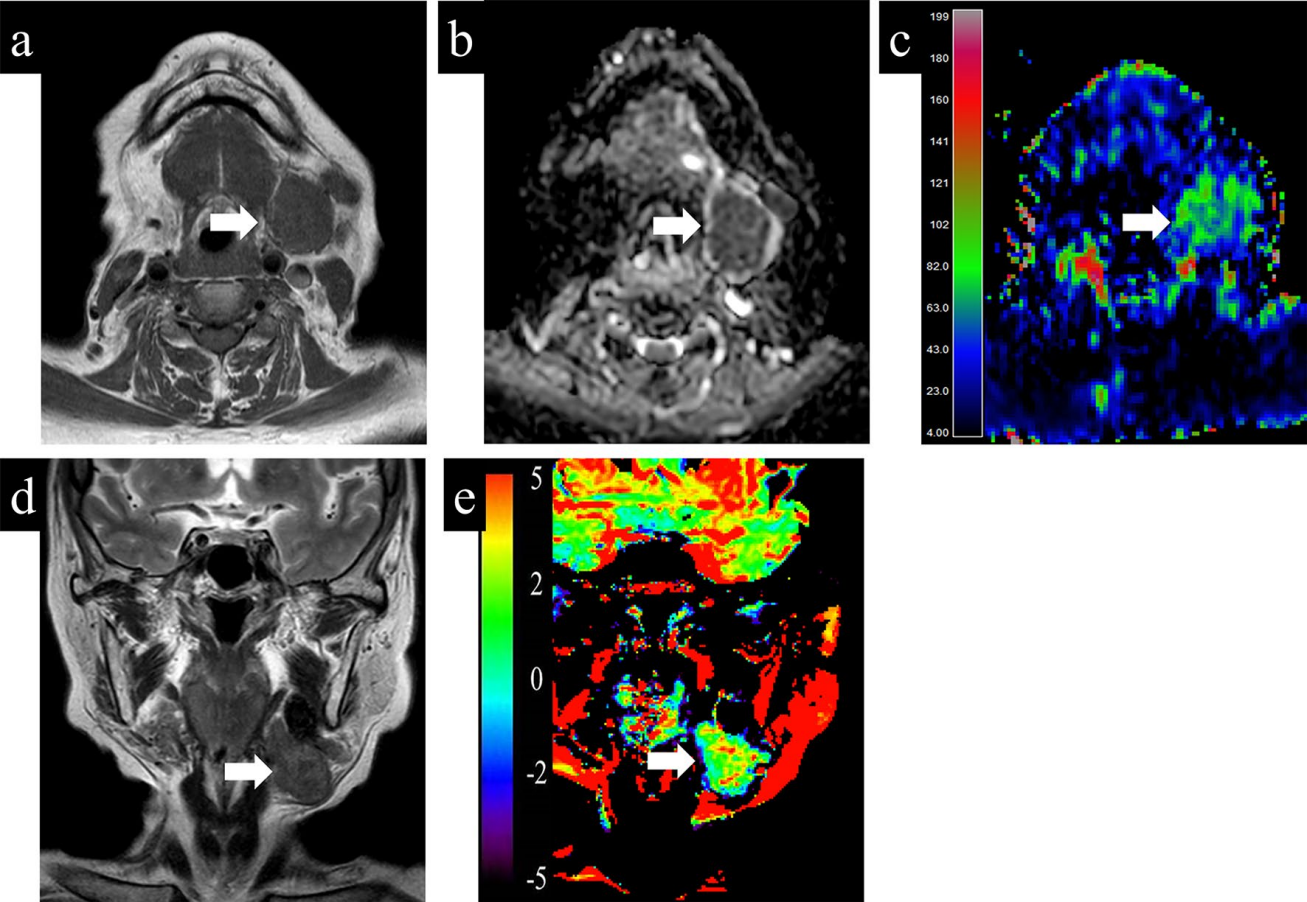
A 75-year-old female with carcinoma ex pleomorphic adenoma. (**a**) T1-weighted image shows a homogeneous low intensity lesion in the left parotid gland. Apparent diffusion coefficient (ADC) (**b**) and tumor blood flow (TBF) (**c**) maps show the lesion with the mean ADC value of 0.91 × 10^−3^ mm^2^/s and the mean TBF value of 67.96 mL/100 g/min, respectively. On T2-weighted coronal image (**d**), the lesion shows slightly higher signal intensity compared to muscle structure. Amide proton transfer (APT) (**e**) map shows the lesion with the mean APT signal intensity of 2.65%. An arrow in each image indicates the lesion.

Representative cases with parotid gland lesions are shown in Figs. 4, 5, 6. Typical pleomorphic adenomas show low TBF, which makes it difficult to distinguish from malignant lesions, while present high ADC and low APT which suggest benign lesions (Fig. 4). On the other hand, Warthin tumors have low ADC, which makes it difficult to distinguish from malignant, while presenting high TBF and low APT, which is consistent as benign (Fig. 5). Most malignant lesions showed high APTSI, low ADC, and moderate TBF (Fig. 6).

## Discussion

This study focused on the diagnostic potential to differentiate benign from malignant salivary gland lesions with a multiparametric non-contrast MR imaging approach, specifically the combination of ADC, TBF and APTSI. The diagnostic performance of the combination of ADC, TBF and APTSI resulted in a significantly increased accuracy compared with each parameter alone. Our study suggests that the combination of ADC, TBF and APTSI can help accurately differentiate malignant from benign salivary gland lesions.

While ADC of malignant salivary gland lesions was significantly lower than that of pleomorphic adenoma, it was not significantly different from overall benign salivary gland lesions including Warthin tumor, which is in line with previous reports^6,8^. Matsushima et al.^23^ reported that the mean ADC of the salivary gland lesions increased with the degree of extracellular components including myxoid and chondroid matrices, microcysts and hyalinization. ADC of pleomorphic adenoma was reported to be significantly higher than that of malignant lesion^7,9,24^, likely reflecting the relative abundance of myxoid and chondroid matrices^24^. On the other hand, with its hypercellular component caused by epithelial proliferation with lymphocytic infiltration^23,25^, Warthin tumor is known to show lower ADC compared to the other benign salivary gland lesions^9,23^, which did not significantly differ from that of malignant lesions^5,7,9^. Thus, ADC does not seem to help differentiate malignant from benign lesions, except for pleomorphic adenomas. In addition, according to a previous report^26^, the reproducibility of ADC values between MR pulse sequences remains controversial. Another report^27^ showed that the ADC value varies depending on the report for differentiating malignant from benign lesion in various organs, and the reproducibility seems to be limited.

The measurement of TBF using ASL has been reported to be useful for diagnosing salivary gland lesions, especially for differentiation of Warthin tumor from the other salivary gland lesions as Warthin tumor has rich vascularity and shows high TBF within the lesion^14^. Congruently, our result showed significantly higher TBF in Warthin tumor than in malignant salivary gland lesions. On the other hand, there was no significant difference in TBF between pleomorphic adenomas and malignant lesions, which is consistent with a previous report^14^.

A recent study reported that the mean APTSI of the malignant parotid lesions was significantly higher than that of the benign parotid lesions^22^. However, their results showed substantial overlap between benign and malignant lesions, resulting in a modest diagnostic accuracy (67.4%). In our result, APTSIs of malignant salivary lesions were significantly higher than those of benign lesions. However, APTSI of Warthin tumor was not significantly different from that of malignant lesions, which is in line with the previous report^22^. Previous studies on brain tumors have demonstrated the positive correlation of APTSI with cellular density and/or Ki-67 labelling^28,29^, which indicated that active proliferation of tumor cells was associated with a high concentration of mobile protein and peptides. The high APTSI of Warthin tumors in our results may be due to high cell density. In addition, some pleomorphic adenomas showed high APTSI in our results, which may be caused by their hypercelluarity variant. Therefore, APTSI alone seems to have a limited diagnostic ability of differentiating benign from malignant lesions.

Multiparametric analysis may be useful to overcome the above-mentioned limitations of single parametric differentiation. However, to date, only few attempts have been made to investigate the utility of multiparametric non-contrast MR imaging approach for salivary gland lesions. A recent study has reported that the combination of TBF and ADC was useful for differentiating pleomorphic adenoma from malignant parotid lesions^12^. However, some pleomorphic adenomas with high cellularity and some Warthin tumor could show low-to-moderate ADC and TBF, which were indicative of malignant lesions^12,14,30,31^. In our results, 4 pleomorphic adenomas and 4 Warthin tumors showed low-to-moderate ADC (≤ 1.516) and TBF (≤ 105.96). Among these, 5 lesions (3 pleomorphic adenomas and 2 Warthin tumors) showed low APTSI (≤ 1.779), which was suggestive of benign lesions. Thus, ADC, TBF and APTSI values may play complementary roles in differentiating malignant from benign salivary gland lesions. To our knowledge, this is the first multiparametric MR imaging study for the salivary gland that included APT-weighted imaging.

The retrospective study design and the relatively small number of malignant lesions are major limitations of this study. A well-designed prospective study with a large number of cases and a wider range of tumor types is needed to confirm our findings. In addition, we did not closely compare histopathological features and imaging findings. Therefore, further investigations are needed to clarify the histopathological characteristics which influenced ADC, TBF and APTSI in salivary gland lesions. Furthermore, this is a single-center study and the results should be compared and verified with those of other institution because differences in vendors, devices, and MR parameters may have a considerable effect on quantitative parameters of this study. We believe our study may encourage future research, and a large multicenter study would be desirable. Finally, all parameters were measured within the ROI avoiding cystic or necrotic parts, would cause selection bias or affect the consistent acquisition of reliable values of each parameter. In addition, in this study, it was not possible to set the ROI in exactly the same shape due to the difference in the image matrix and the direction of the cross section. However, the good interobserver agreement on all parameters proves the reproducibility of our results.

In conclusion, the present study showed that there is a limit to the differentiation of malignant from the benign salivary gland lesions on the basis of ADC, TBF, or APTSI value alone. The combination of ADC, TBF and APTSI can help increase accuracy in differentiating malignant from benign salivary gland lesions.

## Methods

### Patients

This retrospective study was approved by our institutional ethics review board (Ethics Committee on Epidemiological Studies Kagoshima University Graduate School of Medical and Dental Sciences, No. 190303 revised edition 1), and the requirement for informed consent of patients was waived. This study was conducted in accordance with the Declaration of Helsinki and Ethical Guidelines for Medical and Health Research Involving Human Subjects in Japan (https://www.mhlw.go.jp/file/06-Seisakujouhou-10600000-Daijinkanboukouseikagakuka/0000080278.pdf). ASL perfusion imaging and APT imaging have been a part of our routine preoperative MR imaging protocol for head and neck lesions since September 2016. A retrospective review of the MR imaging database and clinical records of our radiology department identified 120 consecutive patients who had undergone pretreatment salivary gland MR examination between September 2016 and December 2019. Among these, 56 patients met the following inclusion criteria: (1) DW, ASL and APT images had been obtained; (2) a pathological diagnosis had been obtained by biopsy or surgical resection. Five patients with predominantly cystic lesions were excluded. Four patients were excluded owing to insufficient image quality related to motion or susceptibility artefacts. We also excluded small lesions (< 10 mm) (n = 5) which were not detected in ASL or APT-weighted imaging. Finally, 42 patients (26 men and 16 women; median age, 65 years; range, 10–89 years) with a total of 42 salivary gland lesions (35 parotid and 7 submandibular gland lesions) were included in this study. Out of the 42 lesions, 31 were benign (12 pleomorphic adenomas, 11 Warthin tumors, 4 IgG4-related disease, one basal cell adenoma, one schwannoma, one Kimura's disease, and one cavernous hemangioma) and 11 were malignant (4 malignant lymphomas, 3 carcinoma ex pleomorphic adenomas, one salivary duct carcinoma, one adenoid cystic carcinoma, one mucoepidermoid carcinoma, and one metastatic lesion from melanoma).

### MR imaging technique

All MR examinations were performed using a 3-T system (Ingenia 3.0T; Philips Healthcare, Best, The Netherlands) with a 20-channel standard phased-array head and neck coil. For minimizing motion artifact, we paid attention to comfortable positioning of patients in the scanner and instruction of the importance of staying still during the scan. In addition, their heads were fixed firmly with the coil to prevent movement during the scan. DW, ASL, and APT-weighted MR images were performed within the same MR examination, and the time interval between these sequences was within about 15 min.

#### DW MR imaging protocols

The DWI was performed in the axial plane with a single-shot spin-echo echo planar imaging sequence with the following parameters: TR, 5000 ms; TE, 85 ms; field of view (FOV), 230 × 230 mm^2^; matrix, 120 × 120; slice thickness, 4 mm; b values = 0 and 800 (s/mm^2^); diffusion gradient direction, 3. The duration of the DWI protocol was about 2 min 25 s. The ADC value was calculated in a voxel-by-voxel manner by mono-exponential fitting with the pair of b-values.

#### ASL perfusion MR imaging protocols

The TBF images were acquired with a three-dimensional pseudo-continuous ASL (3D pCASL) based on volume isotropic turbo spin echo acquisition sequence with the following parameters: slice thickness, 6 mm; number of slices, 15; FOV, 230 × 230 mm^2^; matrix, 80 × 80; TR, 6500 ms; TE, 36 ms; flip angle, 90°; number of excitations, 6; acceleration factor for parallel imaging, 2.5; labeling duration, 3000 ms; postlabeling duration, 1500 ms; and pixel bandwidth, 391.6 Hz/pixel. The position of the labelling plane was located 75 mm below the center of imaging slices. The scanning time was 5 min 25 s.

#### APT-weighted MR imaging protocols

APT-weighted imaging data were acquired in a coronal plane using a single-slice turbo-spin-echo sequence after localized high-order shimming on the slice with the largest lesion cross sectional area. The saturation pulse strength was 2 μT and the duration was 2 s^32^. For the acquisition of the full z-spectrum, the imaging was repeated at 25 saturation frequency offsets from ranging − 6.0 to + 6.0 ppm with a step of 0.5 ppm, as well as one offset far from the water resonant frequency (− 1560 ppm) for signal normalization. Imaging parameters were as follows: FOV, 230 × 230 mm^2^; matrix, 128 × 128; slice thickness, 5 mm; TR, 4000 ms; TE, 4.8 ms; echo train length, 128; number of signal averages, 1; sensitivity encoding factor, 1. δB0 maps were separately acquired with a 2D dual gradient-echo sequence (ΔTE = 1 ms) for a point-by-point δB0 correction^32^. The duration of the APT weighted imaging protocol was 1 min 52 s.

The clinical MR imaging study also included axial T1-weighted turbo spin-echo (TR: 522 ms, TE: 10 ms, FOV: 230 mm, matrix: 336 × 243, slice thickness: 4 mm, gap: 0.8 mm, number of excitations: 1, flip angle: 90, echo train length: 3) and axial T2-weighted turbo spin-echo (TR: 4000 ms, TE: 80 ms, FOV: 230 mm, matrix: 336 × 243, slice thickness: 4 mm, gap: 0.8 mm, number of excitations: 1, flip angle: 90, echo train length: 18).

#### ASL image processing

All ASL images were transferred and analyzed using the interactive data language-based software (Philips Research Integrated Development Environment; Philips Healthcare). We calculated the TBF of 3D pCASL from the signal difference, which was calculated by subtracting the labeled image from the control image using the following formula^33^:

$$\text{TBF }= \frac{6000\cdot \lambda \cdot ({SI}_{control}-{SI}_{label})\cdot {e}^{\frac{PLD}{{T}_{1blood}}}}{2\cdot \alpha \cdot {T}_{1blood}\cdot {SI}_{PD}\cdot (1-{e}^{-\frac{\tau }{{T}_{1blood}}})} [\text{ml}/100\;\;\text{g}/\text{min}]$$

where λ is the blood/tumor-tissue water partition coefficient (1.0 g/mL), SI_control_ and SI_label_ are the time-averaged signal intensities in the control and label images, respectively, T_1blood_ is the longitudinal relaxation time of blood (1650 ms), PLD is postlabeling delay time (1500 ms), α is the labeling efficiency (0.85), SI_PD_ is the signal intensity of a proton density-weighted image, and τ is the label duration (3000 ms).

#### APT-weighted image processing

All APT-weighted images were transferred and analyzed using the software program ImageJ (version 1.48v; National Institutes of Health, Bethesda, MD). The APTSI were obtained by calculating the magnetization transfer ratio asymmetry (MTR_asym_) between ± 3.5 ppm^17^ using the B_0_-corrected MT-spectrum:

$$ {\text{MTR}}_{{{\text{asym}}}} \left( {{3}.{\text{5 ppm}}} \right)  = \left( {{\text{S}}_{{ - {3}.{\text{5 ppm}}}} {-}{\text{ S}}_{{{3}.{\text{5 ppm}}}} } \right) \, /{\text{S}}_{0} \times { 1}00 \, \left( \% \right), $$

where S_±3.5 ppm_ and S_0_ are the signal intensities obtained at ± 3.5 ppm and − 1560 ppm, respectively.

### Imaging analyses

The ADC, TBF and APTSI were measured by two radiologists (K.T. and H.K. with 19 and 3 years, respectively, of experience in radiology) who were blinded to the final pathological results. With reference to T1- and T2-weighted images, the mean values of ADC, TBF and APTSI in each lesion were measured within a region of interest (ROI) delineating the outline of the lesion (mean size, 473.8 ± 636.7 mm^2^, 311.8 ± 340.2 mm^2^, and 294.3 ± 534.3 mm^2^, respectively). Care was taken to avoid cystic or necrotic parts within the lesions.

### Statistical analysis

Interobserver agreement of ADC, TBF, and APTSI measurement was evaluated using intraclass correlation coefficient (ICC) and Bland–Altman plot. ICCs were considered to indicate excellent agreement when > 0.74^34^.

ADC, TBF, and APTSI values of benign and malignant lesions were compared using the Mann–Whitney *U* test. Those parameters were also compared between malignant lesion and each benign subgroup (pleomorphic adenoma or Warthin tumor) using the Mann–Whitney *U* test. ROC curve analysis was performed using the MedCalc version 19.6 (MedCalc Software, Mariakerke, Belgium) to evaluate the ability of each parameter and their combinations to differentiate malignant from benign lesions. For each parameter, optimal cutoff values were chosen by using a threshold criterion that was maximizing the Youden index for predicting malignant lesions. The sensitivity, specificity, positive predictive value, negative predictive value, and accuracy of ADC, TBF, and APTSI for diagnosing malignant lesions were calculated for these individual parameters. Additionally, we examined whether the diagnostic accuracy for malignant lesions could be improved by combining these parameters (ADC and TBF, ADC and APTSI, TBF and APTSI, and combined three parameters [ADC, TBF and APTSI]). Their diagnostic performances were compared by the McNemar test.

All data for continuous variables are presented as mean ± standard deviation (SD). A *P-*value < 0.05 was considered to indicate statistical significance in all analyses. Statistical analyses were performed using MedCalc version 11.1 (MedCalc Software, Mariakerke, Belgium) and SPSS version 23.0 (SPSS, Chicago, IL).

#### Validation study

To test the diagnostic value of the classification method derived from the initial study results, we applied it to 10 consecutive patients with 10 salivary gland lesions who underwent MR examination between December 2019 and December 2020. Out of the 10 lesions, 7 were benign (3 pleomorphic adenomas, 3 Warthin tumors, and one myoepithelioma) and 3 were malignant (one malignant lymphomas, one adenoid cystic carcinoma, and one mucoepidermoid carcinoma). The technique used to evaluate the lesions was the same as that used in the initial study. The sensitivity, specificity, positive predictive value, negative predictive value, and accuracy of the classifications derived from the initial study results were examined.
